# Primary Acute Encephalitis

**Published:** 1891-02-07

**Authors:** 


### PRIMARY ACUTE ENCEPHALITIS.

To primary acute encephalitis, a condition not unknown in adult life, though most frequent in early childhood, Strümpell ascribes some cases of convulsions leading to infantile paralysis, of which embolism, in his opinion, does not afford a sufficient explanation. The usual age is between 1½ and 4 years, and the attack comes on with sudden pain in the head, vomiting, and fever. Sometimes in a few hours, at others not until after some days, convulsions and coma follow. These phenomena gradually pass off, and the child remains perfectly well, but for some permanent effect, as more or less complete paralysis of one limb.

